# Mycorrhizal Fungi *Funneliformis mosseae* Mitigates Cadmium Bioavailability in Pepper Rhizosphere via Glomalin Production and pH Elevation

**DOI:** 10.3390/plants15060952

**Published:** 2026-03-20

**Authors:** Yanlong Jia, Peng Zhou, Dehui Tu, Xiaolong Lan, Wenjie Lin, Dan Xing, Zengping Ning

**Affiliations:** 1 School of Chemistry and Environmental Engineering, Hanshan Normal University, Chaozhou 521041, China; jia-yanlong@hstc.edu.cn (Y.J.);; 2 School of Resources and Environmental Engineering, Guizhou Institute of Technology, Guiyang 550002, China; 3 Institute of Pepper, Guizhou Academy of Agricultural Sciences, Guiyang 550006, China; 4 State Key Laboratory of Environmental Geochemistry, Institute of Geochemistry, Chinese Academy of Sciences, Guiyang 550002, China

**Keywords:** arbuscular mycorrhizal fungi, soil reclamation, metal toxicity, glomalin-related soil protein

## Abstract

Cadmium (Cd) contamination in agricultural soils, especially in regions with a naturally high geochemical background such as Southwest China, poses a serious threat to food safety and the health of terrestrial ecosystems. Although arbuscular mycorrhizal fungi (AMFs) are known to enhance plant tolerance to heavy metals, the specific mechanisms by which dominant AMF species in karst soils—such as *Funneliformis mosseae* (Fm) and *Rhizophagus intraradices* (Ri)—immobilize Cd are not yet fully understood. In this study, a pot experiment with pepper plants was conducted to investigate the effects of Fm and Ri inoculation on Cd geochemistry in both the rhizosphere and bulk soil. Key results showed that AMF inoculation, especially with Fm, significantly reduced total Cd (by up to 33.8%) and bioavailable Cd (by up to 36.3%) concentrations in the soil, with a more pronounced effect within the rhizosphere. Accordingly, Cd content in pepper shoots was reduced by up to 15.0%. Inoculation also increased soil pH, organic matter, available phosphorus, and glomalin-related soil protein (GRSP) content. Redundancy analysis identified soil pH and total extractable GRSP as primary factors negatively correlated with Cd bioavailability. The study concludes that AMFs, particularly Fm, represent a potent bioremediation strategy by effectively immobilizing Cd in contaminated soils through mechanisms linked to GRSP production and pH elevation, thereby reducing its phytoavailability and translocation to edible plant parts.

## 1. Introduction

Globally, approximately 14% to 17% of arable soils are contaminated with toxic metals, posing risks to approximately 900 million to 1.4 billion people living in areas where metal concentrations exceed established health and ecological thresholds [1]. Within this context, heavy metal pollution affects one-fifth of China’s cultivated land, with Cd exhibiting a notably high exceedance rate of 7% at contaminated sites [2]. Cd contamination in agricultural soils not only diminishes crop yields but also leads to its accumulation in edible plant parts, resulting in chronic toxicity in humans following consumption [3]. The excessive and long-term application of chemical fertilizers is a major contributor to Cd contamination in arable soils [4,5,6,7]. Consequently, the utilization of microbial fertilizers has been proposed as a partial substitute for chemical fertilizers, with the dual objective of enhancing soil health and remediating Cd-contaminated lands [8,9,10].

Microorganisms facilitate the remediation of heavy metal pollution not only by transforming, adsorbing, and immobilizing metals through metabolic activities but also by promoting plant nutrient uptake in contaminated environments, thereby augmenting plant stress tolerance and often acting synergistically in phytoremediation strategies [11,12,13,14]. Arbuscular mycorrhizal fungi (AMFs), which form symbiotic associations with most terrestrial plants, play a pivotal role in improving soil quality and represent a key functional microbial group in polluted soils [15]. AMF colonization of plant roots is a well-established strategy to enhance host Cd tolerance. This symbiosis modulates the migration of Cd within the soil–plant system primarily by regulating its uptake and translocation [16,17]. Beyond metal dynamics, AMF inoculation improves plant acquisition of essential minerals such as potassium, phosphorus, and nitrogen, thereby supporting overall plant health under stress [18]. Crucially, studies have consistently shown that AMFs can reduce soil Cd bioavailability and its subsequent uptake by plants [19]. A key mechanism underlying this reduction is the AMF-induced secretion of glomalin-related soil protein (GRSP), which contributes to soil aggregation and metal immobilization, as evidenced by the positive correlation between GRSP content, soil aggregate stability, and reduced Cd mobility observed in inoculation experiments [20]. AMFs influence soil pH and organic matter dynamics through the secretion of chelating agents, including organic acids and GRSP, from their hyphae, thereby altering Cd bioavailability [21,22,23]. GRSP, a glycoprotein secreted by AMFs into the soil [24], exhibits low water solubility and considerable thermal stability. These properties promote soil particle aggregation and structural stability [25], earning it the nickname “biological glue”. These properties underpin its strong affinity for heavy metals, enabling it to modify metal bioavailability and influence plant metal acquisition [26,27]. GRSP can directly adsorb or chelate Cd ions through its abundant functional groups, immobilizing them within hyphal structures or rhizosphere soil to form stable organic Cd complexes. This reduces the concentration of free Cd^2+^ in the soil solution and consequently decreases plant uptake of Cd [28,29].

The karst region of Southwestern China, the world’s largest contiguous karst area, is characterized by naturally elevated background levels of soil Cd [30,31]. This geogenic enrichment, compounded by anthropogenic inputs such as fertilizer application, has resulted in severe Cd contamination in local agricultural soils [32]. Previous research has identified *Funneliformis mosseae* (Fm) and *Rhizophagus intraradices* (Ri) as dominant AMF species in these karst environments [33,34,35]. Nevertheless, the specific mechanisms through which Fm and Ri influence soil Cd bioavailability, particularly the relative contributions of key processes such as GRSP production and rhizosphere pH modulation, remain inadequately elucidated. To address this research gap, the present study investigates the effects of inoculating Cd-contaminated karst soils with these two dominant AMF species. We aim to analyze changes in total Cd, bioavailable Cd, pH, and other critical soil chemical properties, alongside the secretion of GRSP by extraradical hyphae, both within the rhizosphere and in bulk soil. This research seeks to clarify the impact of AMFs on soil Cd dynamics and to delineate the contributions of soil chemical properties and GRSP to Cd immobilization, thereby evaluating the potential of AMFs in the remediation of Cd-contaminated farmland.

## 2. Results

### 2.1. Changes in Soil Cd Content Under the Influence of AMFs

**Figure 1:**
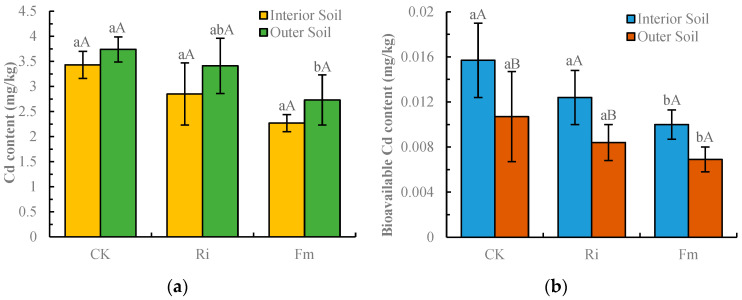
Effects of AMF inoculation on soil Cd content. (**a**) Total Cd content; (**b**) bioavailable Cd content. CK: non-inoculated control; Ri: inoculated with *Rhizophagus intraradices*; Fm: inoculated with *Funneliformis mosseae*. “Interior soil”: soil within a 5 cm radius from the pepper stem (rhizosphere-influenced zone). “Outer soil”: soil near the pot wall (bulk soil zone). Different lowercase letters above the bars indicate significant differences among treatments within the same soil zone (*p* < 0.05). Different uppercase letters indicate significant differences between interior and outer soil within the same treatment (*p* < 0.05).

Microscopic examination confirmed successful root colonization by the respective AMF in inoculated treatments (Fm: 68 ± 5%; Ri: 72 ± 4% root length colonized), while the control treatment showed minimal colonization (<5%). As shown in Figure 1, inoculation treatments significantly decreased both total and bioavailable Cd concentrations in the inner and outer perimeter soils. In Ri-inoculated soils, total Cd was 2.85 mg/kg (inner) and 3.41 mg/kg (outer), with bioavailable Cd at 0.0124 mg/kg and 0.0084 mg/kg, respectively. In Fm-inoculated soils, total Cd was 2.27 mg/kg (inner) and 2.73 mg/kg (outer), with bioavailable Cd at 0.010 mg/kg and 0.0069 mg/kg, respectively. Compared to the control, total Cd in Ri-inoculated soils decreased by 16.4% (inner) and 7.64% (outer), while in Fm-inoculated soils, the decreases were 33.8% (inner) and 27.0% (outer). Similarly, bioavailable Cd reductions were 21.0% (inner) and 20.6% (outer) for Ri, and 36.3% (inner) and 35.5% (outer) for Fm. These results clearly indicate that Fm inoculation is more effective than Ri inoculation in reducing soil total and bioavailable Cd.

### 2.2. Changes in Soil pH and Other Properties Under AMF Influence

Soil samples from the inner and outer perimeters of different treatments were collected and their basic chemical properties were analyzed (Table 1). Significant differences were observed not only among treatments but also between the two zones within the same treatment. Total nitrogen (TN), Total phosphorus (TP), and Total potassium (TK) did not differ significantly between inoculated treatments (Ri and Fm) and the uninoculated control (CK). However, within a given treatment, TP was consistently higher in the outer perimeter soil, with significant differences (*p* < 0.05) in the CK and Fm treatments.

Compared to the CK, Available phosphorus (AP) increased by 59.4% (inner) and 47.0% (outer) in Ri-inoculated soil. In Fm-inoculated soil, AP increased by 72.0% in the inner perimeter but decreased by 9.75% in the outer perimeter. Soil organic matter (OM) content was higher in inoculated treatments, following the order Fm > Ri > CK. Specifically, relative to CK, OM increased by 33.9% (inner) and 5.71% (outer) with Ri inoculation, and by 35.7% (inner) and 9.56% (outer) with Fm inoculation. Moreover, across all treatments, OM was consistently higher in the outer perimeter than in the inner perimeter. Alkaline-hydrolyzable nitrogen (AN) and Available Potassium (AK) generally showed the trend CK > Ri > Fm, although these differences were not statistically significant. Compared to CK, Ri inoculation decreased AN by 6.70% (inner) and 11.5% (outer), and AK by 28.5% (inner) and 23.8% (outer). Fm inoculation decreased AN by 13.4% (inner) and 19.0% (outer), and AK by 52.3% (inner) and 50.3% (outer).

Soil pH in inoculated treatments was slightly higher than in the control. Within each treatment, pH was consistently lower in the inner perimeter than in the outer perimeter soil.

### 2.3. Changes in AM Fungal Secretion of GRSP Content

**Figure 2:**
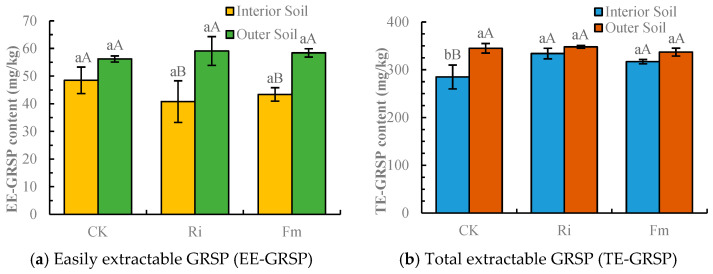
Changes in glomalin-related soil protein (GRSP) content under different AMF inoculation treatments. CK: non-inoculated control; Ri: inoculated with *Rhizophagus intraradices*; Fm: inoculated with *Funneliformis mosseae*. The *Y*-axis unit “mg/kg” represents milligrams of GRSP per kilogram of dry soil. Different lowercase letters above the bars indicate significant differences among treatments within the same soil zone (*p* < 0.05). Different uppercase letters indicate significant differences between interior and outer soil within the same treatment (*p* < 0.05).

As shown in Figure 2, inoculation treatments significantly increased GRSP content. Both easily extractable GRSP (EE-GRSP) and total extractable GRSP (TE-GRSP) varied significantly among treatments. In soils inoculated with Ri, EE-GRSP was 40.8 mg/kg (inner perimeter) and 59.1 mg/kg (outer perimeter), while TE-GRSP was 334 mg/kg and 348 mg/kg, respectively. In soils inoculated with Fm, EE-GRSP was 43.4 mg/kg (inner) and 58.4 mg/kg (outer), and TE-GRSP was 317 mg/kg and 337 mg/kg, respectively. Compared to the control, Ri inoculation decreased inner perimeter EE-GRSP by 15.4% but increased outer perimeter EE-GRSP by 5.82%. Fm inoculation decreased inner EE-GRSP by 9.99% and increased outer EE-GRSP by 4.46%; these changes were not statistically significant. For TE-GRSP, Ri inoculation increased the content by 15.6% (inner) and 1.96% (outer) relative to the control, whereas Fm inoculation increased it by 9.84% (inner) but also decreased it by 1.53% (outer). Both inoculations significantly affected TE-GRSP in the inner perimeter soil. Across all treatments, GRSP content was consistently higher in the outer perimeter soil than in the inner perimeter soil. Overall, Ri inoculation exhibited a more pronounced effect on GRSP content.

### 2.4. Analysis of Factors Influencing Soil Cd Changes Under AMF Influence

We performed a redundancy analysis (RDA) on total soil Cd and bioavailable Cd content with environmental factors to determine the explanatory power of these factors on soil Cd content variations, as shown in Table 2. For the inner perimeter soil, the first and third axes explained 99.1% and 0.88% of the variation in soil Cd content, respectively. In the outer perimeter soil, the first and third axes explained 99.99% and 0.01% of the variation in soil Cd content, respectively. This means that the cumulative explanation of the soil Cd content characteristics by the first and third soil environmental factor axes reached 100%, and their cumulative explanation for the relationship between soil Cd content and environmental factors also reached 100%. This indicates that for both inner and outer perimeter soils, the first and third axes, primarily the first axis, effectively represent the relationship between soil Cd content and environmental factors.

**Figure 3:**
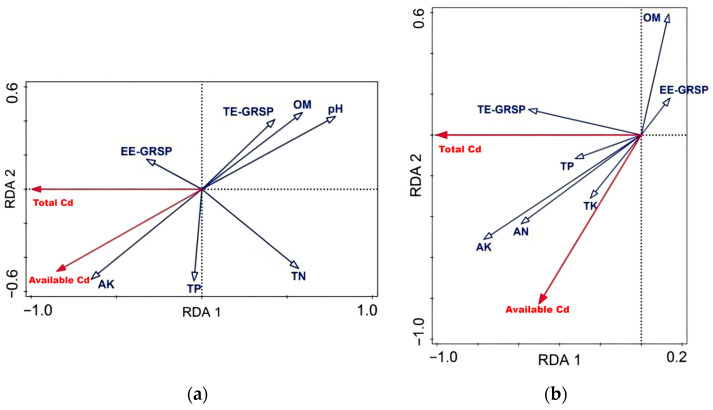
(**a**) Redundancy analysis (RDA) of Cd content and environmental factors for interior soil; (**b**) redundancy analysis (RDA) of Cd content and environmental factors for outer soil. Arrows represent environmental variables; their direction indicates the gradient of the variable’s influence on the ordination, and their lengthis proportional to the strength of that influence. Colors differentiate variable types: red arrowsdenote Cd fractions (Total Cd and Available Cd), blue arrowsdenote soil physicochemical properties and glomalin-related indices. The dashed lines indicate the coordinate axes (the cross quadrants at the origin). TN: Total Nitrogen; TP: Total Phosphorus; TK: Total Potassium; AN: Alkaline-hydrolyzable Nitrogen; AK: Available Potassium; OM: Organic Matter; pH: pH Value; EE-GRSP: Easily Extractable Glomalin-Related Soil Protein; TE-GRSP: Total Extractable Glomalin-Related Soil Protein; Total Cd: Total Cadmium; Available Cd: Bioavailable Cadmium.

The RDA was performed with total Cd and bioavailable Cd as response variables and ten soil environmental factors as explanatory variables: total nitrogen (TN), total phosphorus (TP), total potassium (TK), alkaline-hydrolyzable nitrogen (AN), available phosphorus (AP), available potassium (AK), organic matter (OM), pH, easily extractable GRSP (EE-GRSP), and total extractable GRSP (TE-GRSP) (Figure 3). Forward selection was applied to exclude factors based on the correlation matrix. In the ordination biplot, solid red arrows represent total and bioavailable Cd, while hollow blue arrows represent environmental factors. The angle between an arrow and an axis indicates its correlation with that axis (smaller angle = higher correlation). Arrow length reflects the strength of the correlation between the environmental factor and soil Cd content; longer arrows indicate a stronger influence. A longer perpendicular projection of an environmental factor arrow onto a Cd arrow (i.e., larger cosine value) signifies the greater influence of that factor on Cd content.

The significance test confirmed the reliability of the ordination (first axis and all axes: *p* < 0.05). In the inner perimeter soil (Figure 3a), AK and EE-GRSP showed the smallest angles and aligned in direction with both Cd fractions, indicating a positive correlation. In contrast, TN, OM, TE-GRSP, and pH exhibited angles > 90° with Cd, indicating negative correlations. The pH arrow was the longest, suggesting it had the strongest explanatory power and was the dominant factor influencing soil Cd in the inner perimeter.

In the outer perimeter soil (Figure 3b), TE-GRSP, TP, TK, AK, and AN were positively correlated with Cd, whereas OM and EE-GRSP were negatively correlated. Among these, the AK arrow was the longest, indicating the greatest influence, while the EE-GRSP arrow was the shortest, indicating the weakest influence.

The 2D RDA plot depicts correlations between soil environmental factors and Cd content but does not quantify the precise contribution of each factor. Therefore, we further analyzed the environmental factors using Monte Carlo permutation tests combined with forward selection, with results presented in Table 3. In the inner perimeter soil, pH and TN exhibited the highest explanatory power for soil Cd, accounting for 60.8% and 29.9% of the variation, respectively, indicating they are the most critical factors influencing Cd content. Subsequent factors, in descending order of explanatory power, were TE-GRSP, TP, AK, OM, and EE-GRSP. Notably, only pH, TN, and TE-GRSP showed statistically significant effects (*p* < 0.05), while the influence of the other factors was not significant (*p* > 0.05). In the outer perimeter soil, AK was the dominant factor, explaining 59.4% of the variation in soil Cd content, which was also statistically significant (*p* < 0.05).

### 2.5. Changes in Plant Cadmium Content Under the Influence of AM Fungi

**Figure 4:**
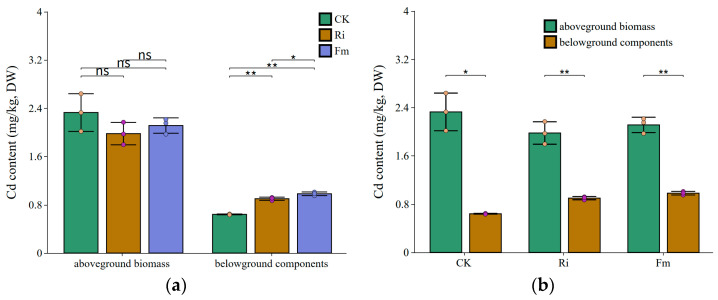
Cadmium (Cd) content in the aerial and belowground parts of chili pepper under different treatments. (**a**) Cd content grouped by plant part (aboveground biomass and belowground components) to compare across inoculation treatments (CK, Ri, Fm). (**b**) Cd content grouped by inoculation treatment to compare between plant parts. CK: non-inoculated control; Ri: inoculated with Rhizophagus intraradices; Fm: inoculated with *Funneliformis mosseae*. The *Y*-axis unit “mg/kg” represents milligrams of Cd per kilogram of plant dry weight. “ns” (not significant) *p* ≥ 0.05; “*” *p* < 0.05; “**” *p* < 0.01.

The distribution of Cd in aerial and belowground tissues of chili pepper under different treatments is presented in Figure 4. In the Ri-inoculated group, Cd content was 1.98 mg/kg in aerial parts and 0.900 mg/kg in belowground parts. In the Fm-inoculated group, the corresponding values were 2.11 mg/kg and 0.982 mg/kg. The CK showed values of 2.33 mg/kg in aerial parts and 0.641 mg/kg in belowground parts. Compared to the CK, Ri and Fm inoculations reduced aerial Cd by 15.0% and 9.25%, respectively, though these reductions were not statistically significant. In contrast, both inoculations significantly increased belowground Cd, with increments of 40.5% for Ri and 53.3% for Fm relative to the CK; the difference between Ri and Fm was also significant. Across all treatments, Cd content in belowground tissues remained significantly lower than in aerial tissues.

## 3. Discussion

### 3.1. Analysis of Changes in Soil Cd Content Within and Outside the AMF Inoculation Zone

The formation of a symbiotic relationship between AMFs and plants enhances Cd sequestration through the strong adsorption capacity of mycorrhizal structures, such as cell walls and vesicles, effectively compartmentalizing Cd at the cellular level [36,37]. In the rhizosphere, the extensive extraradical hyphal network provides a vast surface area, acting as a biological filter for Cd adsorption [26]. Our findings demonstrate that AMF inoculation significantly reduced bioavailable Cd content compared to the non-inoculated control. RDA revealed significant negative correlations between bioavailable Cd and two key factors: soil pH and TE-GRSP. It is critical to interpret these statistical associations as indicators of a multifactorial process. The observed reduction likely results from the integrated effects of several concurrent mechanisms initiated by AMF symbiosis.

Concurrently, AMF secrete metabolites, most notably GRSP, a glycoprotein with a high density of functional groups capable of chelating Cd ions, thereby forming stable complexes that reduce Cd mobility in the soil solution [38,39]. Furthermore, AMF symbiosis can modulate rhizosphere pH. Although hyphal exudation of organic acids may cause localized acidification, a net increase in soil pH, as observed in our study (Table 1), is a commonly reported outcome of AMF colonization. This pH elevation is a critical geochemical driver, as it promotes the adsorption of Cd^2+^ onto soil colloids and favors the formation of less soluble Cd precipitates, thereby directly decreasing the phytoavailable fraction.

The RDA identified soil pH and TE-GRSP as the primary environmental factors negatively correlated with bioavailable Cd concentrations in the rhizosphere (Table 3 and Figure 3a). While correlation does not establish direct causality, it aligns with established mechanisms wherein GRSP chelates and immobilizes Cd ions [28,29] and elevated soil pH promotes Cd adsorption onto soil colloids and the formation of less soluble precipitates [19,40]. The concurrent increase in these factors (pH and GRSP) following Fm inoculation, coupled with the most pronounced reduction in Cd bioavailability, strongly suggests that the combined effect of GRSP-mediated sequestration and pH-driven speciation changes constitutes a primary pathway through which AMFs, particularly Fm, mitigate Cd phytoavailability in this system. It is acknowledged that other unmeasured AMF-induced changes (e.g., alterations in microbial community structure and root exudate profile) may also contribute synergistically.

Conversely, organic acids secreted by AMFs may acidify the inner perimeter soil, potentially resulting in higher bioavailable Cd concentrations relative to the outer perimeter. As illustrated in Figure 1, AMF inoculation reduced total Cd content in both soil zones, with a more pronounced decrease observed in the inner perimeter. This phenomenon may stem from successful AMF colonization altering the rhizosphere environment and root architecture, promoting root tip differentiation and the expansion of root surface area, thereby enhancing Cd absorption efficiency [41,42,43]. Furthermore, extraradical hyphae can adsorb, absorb, and accumulate Cd from the rhizosphere, facilitating its translocation to the host plant and contributing to the overall reduction in soil Cd content [44].

### 3.2. Impact of AMF Inoculation on Soil Chemical Properties and GRSP

AMFs, predominantly inhabiting the root-adjacent soil, exert considerable influence on various soil properties upon inoculation. Colonization of host plants triggers the secretion of H^+^ ions and organic acids by mycorrhizae, potentially lowering rhizosphere pH. This acidification facilitates the dissolution of sparingly soluble inorganic phosphorus, enhancing its plant availability [45]. Subsequent changes in soil pH can, in turn, modulate the distribution of AMFs, further affecting nutrient dynamics.

Both OM and AP significantly impact AMF development. Within a specific range, increased OM content stimulates hyphal branching and growth. In contrast, elevated AP can suppress the formation of vesicles and mycorrhizae by altering nutrient partitioning between the plant and fungus, thereby reducing the allocation of photosynthetic carbon to the AMF [46]. Under high-phosphorus conditions, the inhibition of AMF growth and development leads to hyphal degradation, consequently increasing GRSP release into the soil [47,48]. These mechanisms align with our experimental results, wherein the outer perimeter soils of the CK, Ri, and Fm treatments exhibited higher available phosphorus and correspondingly higher GRSP content compared to the inner perimeter soils.

In this study, TE-GRSP content was marginally higher in AMF-inoculated treatments than in the uninoculated control. This observation is consistent with findings by Bedini et al. [49], who reported similar outcomes following inoculation of *F. mosseae* and *R. intraradices* on alfalfa, reinforcing that AMF colonization elevates soil GRSP. Rillig et al. [50] documented a positive correlation between soil organic carbon, nitrogen, and GRSP, a relationship corroborated by Ma et al. [51], who observed a strong positive correlation between TE-GRSP and soil organic carbon in the rhizosphere of coastal tree species. Our RDA for the inner perimeter soil also indicated a significant positive correlation between TE-GRSP and organic matter, affirming GRSP as a vital component of soil organic carbon [52]. Additionally, GRSP contributes to soil quality and structure by promoting the formation of stable soil aggregates.

### 3.3. Impact of Soil Environmental Factors on Cd Content Under AMFs

AMFs modulate the speciation and bioavailability of heavy metals by altering rhizosphere microbial communities and soil chemistry. A critical relationship exists between soil pH and heavy metal bioavailability; an increase in pH following AMF inoculation has been demonstrated to reduce plant-available metal concentrations [53]. Specifically, within the pH range of 5–7, Cd concentration decreases as pH rises, with acidic conditions favoring enhanced metal bioavailability and plant uptake [54]. This aligns with our experimental findings: the RDA results (Figure 3a) for inner perimeter soil show a significant negative correlation between bioavailable Cd content and pH. Furthermore, OM, TN, and TE-GRSP were positively correlated with pH but negatively correlated with bioavailable Cd.

OM influences heavy metal bioavailability not only through its role in soil fertility but also via complexation reactions between its functional groups and metal ions [55,56]. GRSP can directly immobilize heavy metals or modify their chemical forms, thereby affecting bioavailability. Some studies report a positive correlation between TE-GRSP/EE-GRSP and total soil Cd under Cd stress, alongside a negative correlation with bioavailable Cd [57,58], which contrasts with our findings for the inner perimeter soil. In our RDA, the EE-GRSP vector was relatively short, and EE-GRSP ranked lowest in importance among the factors analyzed in both soil zones (Table 3), indicating a minimal direct influence on Cd dynamics. For the outer perimeter soil, TE-GRSP was positively correlated with total Cd but negatively correlated with bioavailable Cd, whereas in the inner perimeter, it showed a negative correlation with both Cd fractions.

Furthermore, RDA identified AK as a significant factor influencing Cd content. Song et al. [59] observed in a pot experiment that high potassium concentrations markedly increased soil Cd content in the presence of cadmium. However, the precise mechanism underlying potassium’s effect on Cd behavior and its potential interplay with other physiological processes remain poorly understood and warrant further investigation.

### 3.4. AM Fungi Reduce Cd Content in the Aboveground Parts of Pepper

The reduction in Cd content in plant shoots by AMFs involves a synergistic system of interactions among roots, fungi, and soil, centered on fungal-mediated remodeling of the rhizosphere microenvironment and internal Cd allocation [16,60,61]. Our findings provide a clear illustration of this paradigm. Inoculation with AMFs (*Rhizophagus irregularis*, Ri, and *Funneliformis mosseae*, Fm) significantly increased Cd content in the belowground parts of pepper plants (by 40.5% and 53.3%, respectively), while shoot Cd content exhibited a consistent decreasing trend. This distinct pattern—enhanced root Cd sequestration concomitant with reduced shoot Cd accumulation—is a hallmark of AMF-induced phytostabilization and has been robustly documented in diverse plant systems, including *Koelreuteria paniculate* [60], maize [61], wheat [62], and *Lonicera japonica* [27].

At the rhizosphere level, AMFs directly modify the soil environment by secreting metabolites like GRSP and organic acids. GRSP acts as an efficient organic chelator, immobilizing substantial Cd via its functional groups to form stable, insoluble complexes [22,63]. This is supported by our RDA, which indicated an inverse relationship between TE-GRSP and bioavailable Cd. This GRSP-mediated fixation effectively transforms Cd into less labile pools, reducing its phytoavailability [26,57]. Concurrently, AMF hyphae and colonized roots secrete low-molecular-weight organic acids (LMWOAs). While a localized, transient decrease in pH from organic acid secretion could theoretically mobilize some soil-bound Cd, this effect is overwhelmingly counteracted in situ. The extensive hyphal network itself provides an immense surface area for adsorption, and more importantly, the concurrently secreted GRSP network acts as a capture matrix. The net effect, as observed in meta-analyses and mechanistic studies, is a significant reduction in Cd mobility and a decrease in the concentration of free Cd^2+^ ions in the soil solution, thereby diminishing the Cd “pressure” at the root surface [39]. Furthermore, AMFs can elevate rhizosphere pH through mechanisms such as preferential anion uptake, which promotes Cd precipitation (e.g., as CdCO_3_ or Cd_3_(PO_4_)_2_), adding another layer of immobilization [36].

Successful mycorrhizal colonization induces profound morphological and physiological changes in the host root system, fundamentally altering its interaction with Cd [61]. The expanded extraradical hyphal network increases the effective absorptive surface area, which can lead to greater total Cd interception. However, the fate of this intercepted Cd is critically redirected. Intraradical fungal structures, such as arbuscules and particularly vesicles, function as efficient “biological filters” and internal “Cd reservoirs.” This facilitates cellular compartmentalization, a process where Cd is preferentially partitioned into less metabolically active compartments. Subcellular distribution studies in mycorrhizal plants consistently show a higher proportion of Cd allocated to the cell wall fraction (Fcw) and the soluble fraction (Fs, primarily vacuoles) in roots, compared to non-mycorrhizal plants [64,65]. The cell wall, rich in pectins and lignins, whose synthesis can be modulated by AMFs, provides ample cation exchange sites for Cd binding. Meanwhile, AMF symbiosis can upregulate the biosynthesis of phytochelatins and the expression of vacuolar transporter genes (e.g., HMA3), enhancing the formation of Cd–phytochelatin complexes and their sequestration into root vacuoles [27,66]. This multi-layered root sequestration capacity—involving fungal structures, cell wall binding, and vacuolar storage—substantially impedes the loading of Cd into the xylem vessels, the conduit for upward translocation to shoots [40]. Thus, even though the total root Cd content may increase due to enhanced hyphal interception, the translocation factor (TF, shoot Cd/root Cd) is dramatically reduced.

In summary, AMFs employ a dual strategy: externally reducing Cd availability by modulating soil physicochemical properties and internally blocking its translocation through preferential sequestration in root tissues. These pathways act synergistically to significantly reduce Cd accumulation in the edible shoots of pepper while supporting overall plant growth.

It should be noted that this study employed single-species inoculations to isolate and compare the specific effects of Fm and Ri. In natural or applied settings, AMF communities are diverse, and interactions (competition/facilitation) between species could influence the overall remediation outcome, a factor that warrants investigation in future multi-species inoculation studies.

## 4. Materials and Methods

### 4.1. Experimental Materials

The soil used in this study was a yellow soil collected from the Guizhou Academy of Agricultural Sciences, which was not previously contaminated with Cd. Cd was artificially added to achieve a concentration of 3.5 mg/kg. After the addition of Cd solution, the soil was thoroughly mixed, adjusted to 60% of its water-holding capacity, and incubated in the dark at 25 °C for 4 weeks to allow for equilibration/aging of the added Cd before the pot experiment. The air-dried soil exhibited a pH of 5.3, a total nitrogen content of 1.06 g/kg, a total phosphorus content of 2.06 g/kg, and a total potassium content of 14.3 g/kg. The chili pepper variety used in the experiment was “Lay an 201,” provided by the Chili Pepper Research Institute, Guizhou Academy of Agricultural Sciences. Chili pepper (*Capsicum annuum* L.) seeds of the variety “Lay an 201” were surface-sterilized with 2% (*v*/*v*) sodium hypochlorite for 10 min, rinsed thoroughly with sterile distilled water, and germinated on moist filter paper in Petri dishes at 25 °C in the dark. After 7 days, uniformly germinated seedlings were transplanted into sterilized seedling trays containing a peat-based substrate and grown for an additional 3 weeks under controlled greenhouse conditions (25/20 °C day/night, 16 h photoperiod, and 60% relative humidity) to obtain healthy seedlings for the pot experiment. The microbial inoculants used were Fm and Ri, maintained by the Microbial Laboratory at the Institute of Plant Nutrition and Resources, Beijing Academy of Agriculture and Forestry Sciences. For this study, the inocula were propagated in a pot culture system using sterile river sand as the substrate with white clover (*Trifolium repens*) and maize (*Zea mays*) as successive host plants for 4 months. The resulting inoculum consisted of spores, extraradical hyphae, infected root fragments, and the substrate. The spore density was quantified as 112–137 spores per 10 g of inoculant (wet weight) for both species using the wet sieving and decanting method.

### 4.2. Experimental Design

A pot experiment with three treatments was established in a completely randomized design with three biological replicates per treatment: (1) CK (non-inoculated control): plants inoculated with 50 g of autoclaved mixed inoculum (Fm and Ri); (2) Ri Treatment: plants inoculated with 50 g of active Rhizophagus intraradices inoculum; (3) Fm Treatment: plants inoculated with 50 g of active Fm inoculum. The use of autoclaved inoculum in the CK controlled for the effects of adding non-living organic matter and substrate associated with the inoculant. Each plastic pot (top diameter: 18.7 cm; bottom diameter: 15.0 cm; height: 19.2 cm) was filled with 5 kg of sterilized Cd-contaminated soil. To prevent cross-contamination between treatments, each pot was considered an independent experimental unit and was placed on a separate saucer. There was no physical connection between pots. For the inoculation treatments, at the time of seedling transplanting, 50 g of the respective fresh inoculum (Fm or Ri) was placed in a 5 cm deep planting hole, ensuring direct contact between the pepper roots and the inoculum. For the non-inoculated control (CK), 50 g of autoclaved (121 °C, 1 h, twice) inoculum (a 1:1 mixture of sterilized Fm and Ri inoculum) was applied in an identical manner to standardize the introduction of non-living organic matter and microbial remnants across all treatments.

**Figure 5:**
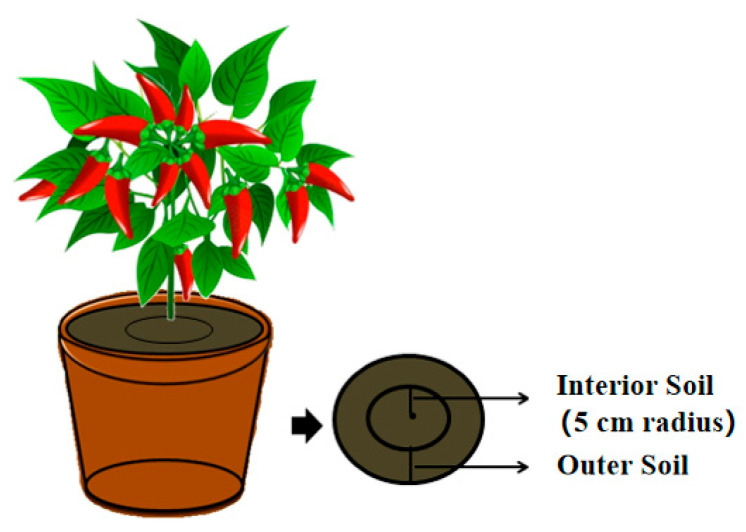
Schematic diagram of inner and outer soil sampling zones. The arrows indicate the location of the Interior Soil (within a 5 cm radius from the stem base) and Outer Soilzones.

After transplantation, all pots were placed in a controlled-environment growth chamber. The conditions were maintained at 25/20 °C (day/night), with a 16 h photoperiod provided by a combination of metal halide and high-pressure sodium lamps, delivering a photosynthetic photon flux density (PPFD) of 400–500 μmol m^−2^ s^−1^ at the canopy level. Relative humidity was maintained at 60–70%. Plants were watered regularly with a quantitative amount during the growth period. No additional fertilizers were applied during the experiment. After 90 days of growth, the chili peppers were harvested, and soil samples were collected from each pot. Soil was sampled from two distinct zones: an inner zone within a 5 cm radius circle centered around the base of the chili pepper plant and an outer zone near the pot wall (Figure 5).

### 4.3. Analytical Parameters and Methods

#### 4.3.1. Soil Chemical Property Determination

Soil chemical properties were determined following the soil agrochemical analysis methods outlined by Bao Shidan [67]. Specifically, the pH value was measured using a glass electrode method with a soil-to-water ratio of 1:2.5. OM was determined by the potassium dichromate volumetric method. TN was determined using the Kjeldahl digestion method, followed by measurement with a Kjeldahl nitrogen analyzer (model KT8000, FOSS, Hillerød, Denmark). TP was determined after HClO_4_-H_2_SO_4_ digestion (with HF removal) and measured using an enzyme-linked immunoassay plate reader (Thermo Fisher Multiskan GO1510, Waltham, MA, USA). TK was determined after HF-HClO_4_ digestion and measured using a flame photometer (model FP6410, Shanghai Yidian Analytical Instrument Co., Ltd., Shanghai, China). AN was measured using the alkaline hydrolysis diffusion method. AP was determined by sodium bicarbonate extraction followed by the molybdenum–antimony anti-colorimetric method.

#### 4.3.2. Determination of GRSP

GRSP was determined according to a slightly modified method based on Wright [68]. The specific procedure was as follows:

EE-GRSP: Weigh 0.5 g of air-dried soil, sieved to less than 2 mm, into a 10 mL centrifuge tube. Add 4 mL of sodium citrate extracting solution (20 mmol/L; pH 7.0) and mix thoroughly. Place the mixture in an autoclave at 121 °C and 0.1 MPa for 30 min. Immediately centrifuge at 10,000 rpm for 6 min, ensuring centrifugation is completed within 30 min. Collect the reddish-brown supernatant and store at 4 °C. EE-GRSP concentration was determined using the Bradford method.

TE-GRSP: Weigh 0.5 g of air-dried soil, sieved to less than 2 mm, into a 10 mL centrifuge tube. Add 4 mL of sodium citrate extracting solution (50 mmol/L; pH 8.0) and mix thoroughly. Place the mixture in an autoclave at 121 °C and 0.1 MPa for 90 min. Centrifuge at 10,000 rpm for 6 min and collect the supernatant in a 50 mL centrifuge tube. Add an equal volume of sodium citrate extracting solution to the centrifuge tube again and extract for 60 min at varying temperatures. Centrifuge and transfer the supernatant. Repeat this process, performing successive extractions until the supernatant no longer appears reddish-brown. Store the collected solution at 4 °C. TE-GRSP concentration was determined using the Bradford method.

#### 4.3.3. Determination of Soil Cd Content

Total Cd in soil was determined by weighing 0.1000 g of the soil sample into a digestion tube. Then, 4 mL of HNO_3_, 2 mL of HClO_4_, and 3 mL of HF were added. The mixture was digested in a digestion furnace until the sample was completely dissolved, and then acid was driven off until the solution was reduced to the size of a soybean, ensuring no HF residue remained. Finally, the solution was brought to a final volume of 50 mL with ultrapure water, and the Cd content was measured using ICP-MS (Thermo Fisher Scientific X2, Thermo Fisher Scientific, Waltham, MA, USA). For bioavailable Cd, 3.00 g of soil sample was weighed into a 50 mL centrifuge tube. Then, 30 mL of 0.01 mol/L CaCl_2_ solution was added, and the mixture was shaken for 2 h (220 r/min). Subsequently, the suspension was centrifuged at 5000 r/min for 5 min and then filtered. The Cd content in the filtrate was measured using ICP-MS (Thermo Fisher Scientific X2, Thermo Fisher Scientific, Waltham, MA, USA).

#### 4.3.4. Assessment of Arbuscular Mycorrhizal Colonization

At harvest, fine root subsamples from each plant were collected, cleared in 10% KOH, and stained with 0.05% trypan blue in lactoglycerol according to the method of Phillips and Hayman (1970) [69]. The percentage of root length colonized by AMF structures (hyphae, arbuscules, and vesicles) was estimated using the grid-line intersection method under a compound microscope at 200× magnification. For each sample, at least 100 intersections were examined. Only treatments inoculated with live Fm or Ri showed significant colonization (>60% root length colonized), confirming the successful establishment of the symbiosis. The CK treatment showed negligible colonization (<5%).

### 4.4. Data Processing and Statistical Analysis

Data were recorded and organized using Excel 2010. Duncan’s multiple range test was performed using DPS 18.10 to determine significant differences among treatment means at *p* < 0.05. Figures were generated using Origin 9.1 software. RDA was performed using Canoco 5 software to explore the relationships between soil Cd fractions (total Cd and bioavailable Cd as response variables) and environmental factors (as explanatory variables). The environmental factor data were log(x + 1)-transformed to improve normality and homoscedasticity. Forward selection with Monte Carlo permutation tests (999 permutations) was applied to identify the significant explanatory variables (*p* < 0.05). The results of the RDA are presented in the ordination biplot (Figure 3) and the associated variance partitioning tables (Table 2 and Table 3).

## 5. Conclusions

This study found that AMF inoculation, especially with Fm, significantly decreased both total and bioavailable Cd in soil and reduced Cd accumulation in pepper shoots. Fm was more effective than Ri, yielding the lowest soil Cd levels (e.g., 2.27 mg/kg total Cd in the inner zone). The reduction was achieved by reshaping the rhizosphere (e.g., increasing pH and GRSP) and enhancing root Cd retention. Redundancy analysis linked TE-GRSP, but not EE-GRSP, to soil Cd. Key influencing factors differed spatially: pH and TN dominated in the inner zone, while AK and TE-GRSP were primary in the outer zone. AMFs altered Cd distribution in plants, promoting root sequestration, likely via vacuolar compartmentalization, thereby lowering Cd in edible parts.

While promising, the efficacy of Fm requires validation under diverse field conditions. Key challenges include the specificity of plant–fungus–soil interactions, the long-term stability of Cd immobilization, and potential Cd remobilization. Future studies are warranted to: (1) use isotopic and molecular methods to quantify root Cd sequestration mechanisms; (2) conduct long-term field trials to assess agronomic and ecological safety; (3) explore synergies between AMFs and other amendments (e.g., biochar) for sustainable remediation.

## Figures and Tables

**Table 1:** Soil chemical properties.

Soil Chemical Properties	CK		Ri		Fm	
	Interior Soil	Outer Soil	Interior Soil	Outer Soil	Interior Soil	Outer Soil
TN (g/kg)	1.41 ± 0.14 aA	1.57 ± 0.39 aA	1.29 ± 0.20 aB	1.68 ± 0.27 aA	1.47 ± 0.06 aB	1.84 ± 0.05 aA
TP (g/kg)	1.94 ± 0.13 aB	3.15 ± 0.46 aA	2.09 ± 0.52 aA	2.62 ± 0.10 aA	1.76 ± 0.43 aB	2.81 ± 0.30 aA
TK (g/kg)	9.49 ± 0.94 aA	9.61 ± 0.20 aA	9.94 ± 0.40 aA	9.62 ± 0.33 aA	8.43 ± 0.71 bA	9.41 ± 0.56 aA
AN (mg/kg)	83.9 ± 18.6 aA	100 ± 10.7 aA	78.3 ± 4.88 aA	88.9 ± 3.42 abA	72.7 ± 6.41 aA	81.3 ± 3.03 bA
AP (mg/kg)	25.9 ± 2.73 aB	50.3 ± 20.3 bA	41.3 ± 2.15 aB	73.9 ± 20.7 aA	44.6 ± 1.76 aA	45.4 ± 3.28 bA
AK (mg/kg)	222 ± 22.5 aB	294 ± 19.7 aA	159 ± 30.8 bA	224 ± 39.2 bB	106 ± 15.7 cA	146 ± 19.1 cA
OM (g/kg)	32.5 ± 9.43 bB	50.2 ± 4.53 aA	43.6 ± 1.08 aB	53.1 ± 2.20 aA	44.1 ± 2.64 aB	55.0 ± 3.69 aA
pH	5.29 ± 0.02 cB	5.35 ± 0.05 cA	5.36 ± 0.03 bB	5.42 ± 0.01 bA	5.46 ± 0.01 aB	5.54 ± 0.02 aA

TN: Total Nitrogen; TP: Total Phosphorus; TK: Total Potassium; AN: Alkaline-hydrolyzable Nitrogen; AP: Available Phosphorus; AK: Available Potassium; OM: Organic Matter. Data are means ± SE (*n* = 3). CK: non-inoculated control; Ri: inoculated with *Rhizophagus intraradices*; Fm: inoculated with *Funneliformis mosseae*. Different lowercase letters indicate significant differences among treatments within the same soil zone (*p* < 0.05). Different uppercase letters indicate significant differences between interior and outer soil within the same treatment (*p* < 0.05).

**Table 2:** Cumulative explained variance of soil Cd content in RDA ordination.

Inoculated Zone	Ordination Axis	Explanatory Power of Soil Cd Content	Correlation of Soil Cd Content with Environmental Factors	Cumulative Explained Variance of Soil Cd Content	Cumulative Explained Variance of the Soil Cd–Environment Relationship
Interior Soil	First Axis	99.12	0.9956	99.12	100.00
	Second Axis	0.00	0.9652	99.12	100.00
	Third Axis	00.88	0.0000	100.00	
	Fourth Axis	00.00	0.0000		
Outer Soil	First Axis	99.99	1.0000	99.99	100.00
	Second Axis	00.00	0.9550	99.99	100.00
	Third Axis	00.01	0.0000	100.00	
	Fourth Axis	00.00	0.000		

**Table 3:** Ranking of soil environmental factor importance and significance test results.

Inoculated Zone	Environmental Factors	Priority Ranking	Explained Variance (%)	F-Value	*p*-Value
Interior Soil	pH	1	60.8	10.9	0.02
	TN	2	29.9	19.2	0.01
	TE-GRSP	3	6.7	12.6	0.02
	TP	4	0.5	0.8	0.39
	AK	5	0.4	0.7	0.418
	OM	6	0.5	0.8	0.478
	EE-GRSP	7	0.4	0.5	0.596
Outer Soil	AK	1	59.4	10.3	0.022
	TE-GRSP	2	10.3	2.0	0.188
	AN	3	4.9	1.0	0.326
	TP	4	5.5	1.1	0.342
	OM	5	2.4	0.4	0.578
	TK	6	12.0	4.4	0.176
	EE-GRSP	7	5.5	0.4	0.064

## Data Availability

The datasets generated during and/or analyzed during the current study are available from the corresponding authors on reasonable request.

## Abbreviations

The following abbreviations are used in this manuscript

AMF	Arbuscular Mycorrhizal Fungus
Fm	*Funneliformis mosseae*
Ri	*Rhizophagus intraradices*
GRSP	Glomalin-Related Soil Protein
EE-GRSP	Easily Extractable Glomalin-Related Soil Protein
TE-GRSP	Total Extractable Glomalin-Related Soil Protein
TN	Total Nitrogen
TP	Total Phosphorus
TK	Total Potassium
AN	Alkaline-hydrolyzable Nitrogen
AK	Available Potassium
OM	Organic Matter
RDA	Redundancy Analysis
